# Multivalent Display of SARS-CoV-2 Spike (RBD Domain) of COVID-19 to Nanomaterial, Protein Ferritin Nanocages

**DOI:** 10.3390/biom11020297

**Published:** 2021-02-17

**Authors:** Umesh Kalathiya, Monikaben Padariya, Robin Fahraeus, Soumyananda Chakraborti, Ted R. Hupp

**Affiliations:** 1 University of Gdansk, International Centre for Cancer Vaccine Science, ul. Kładki 24, 80-822 Gdansk, Poland; monikaben.padariya@ug.edu.pl (M.P.); robin.fahraeus@inserm.fr (R.F.); 2 National Institute of Malaria Research, Dwarka, New Delhi 110077, India; 3 Institute of Genetics and Molecular Medicine, University of Edinburgh, Edinburgh, Scotland EH4 2XR, UK

**Keywords:** ferritin nanocage, SARS-CoV-2, COVID-19, spike, vaccine, receptor-binding domain (RBD), molecular dynamic simulation, ACE2, protein–protein interaction, hydrogen bonds

## Abstract

SARS-CoV-2, or COVID-19, has a devastating effect on our society, both in terms of quality of life and death rates; hence, there is an urgent need for developing safe and effective therapeutics against SARS-CoV-2. The most promising strategy to fight against this deadly virus is to develop an effective vaccine. Internalization of SARS-CoV-2 into the human host cell mainly occurs through the binding of the coronavirus spike protein (a trimeric surface glycoprotein) to the human angiotensin-converting enzyme 2 (ACE2) receptor. The spike-ACE2 protein–protein interaction is mediated through the receptor-binding domain (RBD) of the spike protein. Mutations in the spike RBD can significantly alter interactions with the ACE2 host receptor. Due to its important role in virus transmission, the spike RBD is considered to be one of the key molecular targets for vaccine development. In this study, a spike RBD-based subunit vaccine was designed by utilizing a ferritin protein nanocage as a scaffold. Several fusion protein constructs were designed in silico by connecting the spike RBD via a synthetic linker (different sizes) to different ferritin subunits (H-ferritin and L-ferritin). The stability and the dynamics of the engineered nanocage constructs were tested by extensive molecular dynamics simulation (MDS). Based on our MDS analysis, a five amino acid-based short linker (S-Linker) was the most effective for displaying the spike RBD over the surface of ferritin. The behavior of the spike RBD binding regions from the designed chimeric nanocages with the ACE2 receptor was highlighted. These data propose an effective multivalent synthetic nanocage, which might form the basis for new vaccine therapeutics designed against viruses such as SARS-CoV-2.

## 1. Introduction

The worldwide pandemic caused by SARS-CoV-2 is a very serious threat to public health. So far, there have been over 95.6 million confirmed cases of COVID-19, and over 2.04 million casualties reported worldwide [1]. Several strategies have emerged since its outbreak to control this deadly virus; however, the most promising strategy and long-term solution is to develop an effective vaccine. Vaccine scaffolds can be of different types; however, for the SARS-CoV-2 treatment, protein subunit vaccines and genetically encoded nucleic acid vaccines are the most effective [2].

**Figure 1:**
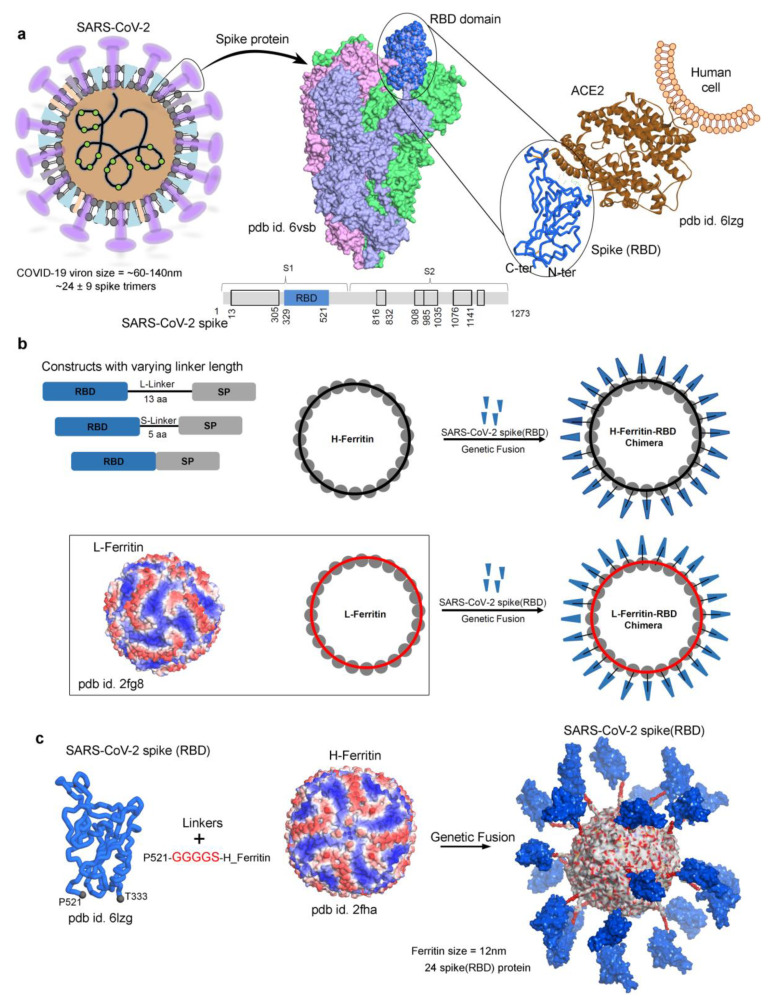
The construct of a SARS-CoV-2 spike receptor-binding domain (RBD) protein over the surface of a ferritin nanocage. (**a**) Spike proteins derived from the COVID-19 viron. A single viron’s size can be ~60–140 nm and consist of ~24 ± 9 spike homotrimers [29,37,38,39]. The crystal structures of a SARS-CoV-2 spike in its homotrimer state (pdb id. 6vsb [3]), as well as the monomeric spike RBD with the human angiotensin-converting enzyme 2 (ACE2) receptor (pdb id. 6lzg [34]), are presented. The spike RBD (residue range 329–521) from the SARS-CoV-2 spike protein is shown in blue. (**b**) A cartoon representation of different chimeric constructs with varying lengths of linkers (No-Linker; 5 aa S-Linker, GGGGS; and 13 aa L-Linker, GGGSGGGGSGGGS), displaying the spike RBD monomers over two different ferritin nanocages (L- and H-ferritin). The individual subunits of ferritin (total twenty-four), represented as spheres, are connected to the RBD domain of the spike protein (magenta triangle) via the linker (black line). For the modeling of the L-ferritin nanocage, the crystal structure (pdb id. 2fg8 [33]) was used, and the cage was constructed using the Proteins, Interfaces, Structures and Assemblies (PDBePISA) server [40]. (**c**) An example of one of the spike RBD–ferritin nanocage systems (5 aa S-Linker chimeric construct). The spike RBD domain (24 monomers; pdb id. 6lzg [34]) is presented over the H-ferritin (pdb id. 2fha [32]), where the size of the H-ferritin cage is 12 nm (outer diameter) and 8 nm (inner diameter) [3,29,37,39].

Structurally, SARS-CoV-2 is a classic viral nanostructure consisting of nuclear material (RNA genome) surrounded by several (four) coat proteins including the SARS-CoV-2 spike (S) glycoprotein (Figure 1) [3]. Among different coat proteins from SARS-CoV-2, the spike protein is the most crucial for the host angiotensin-converting enzyme 2 (ACE2) receptor binding [4]. Structurally, the spike protein is homotrimeric in nature, and the protein is also glycosylated. During translation, the spike protein is generated as a single polypeptide chain though later processed (cleaved) to form two subunits, S1 and S2, respectively (Figure 1) [3]. The S1 fragment of the spike protein binds the ACE2 receptor through utilization of a 25 kDa receptor-binding domain (RBD; residue range 329–521; Figure 1) [5,6]. Several studies demonstrated that the RBD domain of the spike protein forms a therapeutic target of SARS-CoV-2 [7,8].

In recent years, there has been tremendous progress in the protein-based nanomaterial field. Among different protein-based nanomaterials, protein nanocages are perhaps the most sophisticated [9]. Their self-assembly from a small number of subunits into symmetrical, monodispersed architectures has inspired scientists from diverse disciplines [10,11]. In the last two decades, protein nanocages have developed as extremely useful materials for a variety of applications including vaccine development, mostly because of their remarkable diversity in size, shape, structural biocompatibility, and immunogenicity [11,12,13]. In general, protein cages can be viewed as macromolecular containers with a wide range of cargo encapsulation and displaying abilities [14,15,16]. Among different protein-based nanocages, ferritin was the first protein cage isolated, characterized, and found very useful for a number of applications [17]. The physiological function of ferritin is iron storage, and the protein is found abundantly among all organisms except yeast [18]. Ferritin in general is found to be extremely stable (thermostable and protease-resistant) and biocompatible [19]. The outer and inner diameters of ferritin cages are 12 and 8 nm, respectively, and they also carry a central cavity to store iron [16]. One of the reasons ferritin is so useful for biological applications is because the surfaces of ferritin, including the inner, outer, and inter-subunit interfaces, are amenable to different types of modifications [20,21].

Ferritin-based nanocages have emerged as an attractive platform for vaccine generation [17]. In addition, ferritin is capable of a multivalent display of antigen molecules, and a multivalent presentation of antigens generally elicits a relatively potent immune response [22]. These ferritin nanocages were used to display antigens from different pathogens, including influenza [23,24], HIV-1 [25], Lyme disease (OspA) [26], Epstein–Barr virus [27], and respiratory syncytial virus [28], which produces a very high immune response in almost all cases. Additionally, ferritin-based vaccines were also used in humans in two separate clinical trials [23,29]. H-ferritin and *Helicobacter pylori* ferritin (Hp-ferritin), which structurally and functionally closely resemble each other, are the two most common ferritin scaffolds used for antigen display [23,24,25,26,27,28]. Hp-ferritin has been primarily used for trimeric antigen display [23,25,28], whereas H-ferritin has been mostly used for monomeric antigen display [24].

The influenza and SARS-CoV-2 viruses are related in terms of their structure and infectivity (i.e., both affecting the upper respiratory system) [28]. As the ferritin-based vaccine platform already shows a promising immune response against influenza [24], we propose that the ferritin-based nanoparticle platforms would be equally effective in RBD display. In fact, recent studies verified that when the RBD domain (from different proteins) and ferritin were stitched together using variable linkers (genetic fusion), the synthetic chimeras displaying the RBD domain were effective in eliciting moderate to high immune responses in experimental animal models [29,30,31]. However, no high-resolution structures of these synthetic chimeras are available, and we lack details on the dynamics of these highly versatile systems.

Focusing on the SARS-CoV-2 virus, an open question exists as to how novel chimeric RBD nanocages would interact with the ACE2 receptor, and how different linkers fusing the RBD domain with the cage would alter such protein–protein interactions. In order to bring insight into these nanocage systems, we in silico designed (replicated) several spike RBD–ferritin synthetic proteins (utilizing two different ferritin systems—H-ferritin [32] and L-ferritin [33])—with variable linkers (a No-Linker, a small(S)-Linker with 5 amino acids (GGGGS), and a large(L)-Linker with 13 amino acids (GGGSGGGGSGGGS)) and performed an extensive molecular dynamics simulation (MDS). The findings from this study suggest that synthetic SARS-CoV-2 spike RBD–ferritin nanocages are highly dynamic in nature, and determined the optimum length of the linker that is necessary for holding the antigen molecule safely. We also compared the difference in dynamics between two different ferritins when they carried the same antigen molecules (spike RBD domain). In addition, the behavior of the spike RBD regions (470TEIYQAGSTPCNGVEGFNCYF490 and 498QPTNGVGY505 [34,35,36]) responsible for interacting with the host ACE2 receptor was explicitly evaluated. To our knowledge, this is the first comprehensive computational study showing the dynamics of ferritin–RBD constructs in detail, which might have an impact on future vaccine development against SARS-CoV-2 and/or related coronaviruses.

## 2. Materials and Methods

The crystal structures for H-ferritin (pdb id. 2fha [32]) and L-ferritin (pdb id. 2fg8 [33]) in their monomer forms are available in the protein data bank (PDB) database (www.rcsb.org) (Figure 1b,c). The 24 monomers of H- and L-ferritin were constructed/assembled together to form a protein nanocage using the Proteins, Interfaces, Structures and Assemblies (PDBePISA) server [40]. The SARS-CoV-2 spike RBD (pdb id. 6lzg [34]; residue range T333-P521) crystal structure in its active form (“up” conformation; Figure 1) binding to ACE2 receptor was considered to present 24 spike RBD monomers over the ferritin nanocage. The amino acid coordinates for the 5 aa S-Linker (GGGGS) and 13 aa L-Linker (GGGSGGGGSGGGS) linkers were built/modeled using the molecular operating environment (MOE; Chemical Computing Group Inc., Montreal, QC, Canada) package [41,42]. After modeling the required structures of the cage, linkers, and spike RBD, they were further assembled together using the MOE package (Chemical Computing Group Inc., Montreal, QC, Canada). In addition, each merged cage-linker-spike RBD complex was processed through energy minimization using the MOE package (Chemical Computing Group Inc., Montreal, QC, Canada) and applying the CHARMM27 force field [43] to equilibrate the structures. The complete M2e [24,44] protein structure was built using the Phyre2 server [45], and 24 individual monomers of M2e (residue range M1-D72) were presented over the ferritin nanocage using the MOE package (Chemical Computing Group Inc., Montreal, QC, Canada). The ferritin nanocage systems successfully generated and used for MD simulations were as follows: (i) Spike RBD-H_ferritin, (ii) Spike RBD-GGGGS-H_ferritin, (iii) Spike RBD-GGGSGGGGSGGGS-H_ferritin, (iv) 3M2e_GGGGS-H_ferritin, (v) Spike RBD-L_ferritin, and (vi) Spike RBD-SGGGG-L_ferritin. For the spike RBD–ferritin constructs in particular, the starting configurations of all three (linkers) cases simulated were given specific grafting sites on the ferritin nanocage surface (Figure 1c and Supplementary Materials Videos S1–S3). These grafting sites were distributed in a manner to maximize the distances between the spike RBD monomers over the nanocage, and to avoid the inter-spike RBD as well as the spike RBD–nanocage interactions at the initial time steps.

The extensive molecular dynamics simulations on the six modeled systems were performed using the GROMACS 4.6.5 [46,47] package, by applying the CHARMM27 force field [43]. Each individual spike RBD/M2e-ferritin complex was placed in the center of a dodecahedron simulation box, and the system was solvated by single-point charge (SPC) water molecules. The distance between any atom of the protein structure and the boundary of the dodecahedron box was kept at a minimum of 10 Å (thick). Periodic boundary conditions were applied in all directions, and the Na+ and Cl- counter ions were added to produce neutral systems (to match a physiological salt concentration of 150 mM). The steepest descent algorithm was used to minimize the total potential energy of each system, or until the local minimum was obtained, with the equilibration time step set to 50,000. The particle mesh Ewald (PME) method [48] was employed to treat the long-range electrostatic interactions, and the bonds containing hydrogen atoms were constrained using the LINCS (LINear Constraint Solver) algorithm [49]. The cutoffs for the electrostatic (Coulomb) and van der Waals interactions were set to 10 Å. Each system was subsequently equilibrated using the NPT (number of particles (N), system pressure (P), and temperature (T); isobaric-isothermal) ensemble simulation for 1000 ps. The standard temperature and pressure were set to 300 K and 1 bar, respectively, and maintained by applying the V-rescale thermostat [50] and the Parrinello–Rahman pressure coupling method [51], respectively. Equations of motion were integrated using the leapfrog integrator [52], and the atom coordinates were saved every 10 ps. All systems were simulated, or the production run was performed for 100 ns (50 million time steps) and analyzed using the GROMACS package and visual molecular dynamics (VMD) [53] tools. The hydrogen bond interactions were defined on the basis of the donor-acceptor distance being smaller than 3.5 Å and the donor–hydrogen–acceptor angle being 160°–180°. The MOE (Chemical Computing Group Inc., Montreal, QC, Canada), the BIOVIA Discovery Studio (Dassault Systèmes, BIOVIA Corp., San Diego, CA, USA), and VMD tools [53] were used for the visualization of the protein structures, for generating graph plots, and for tracing different types of interactions.

## 3. Results and Discussion

**Figure 2:**
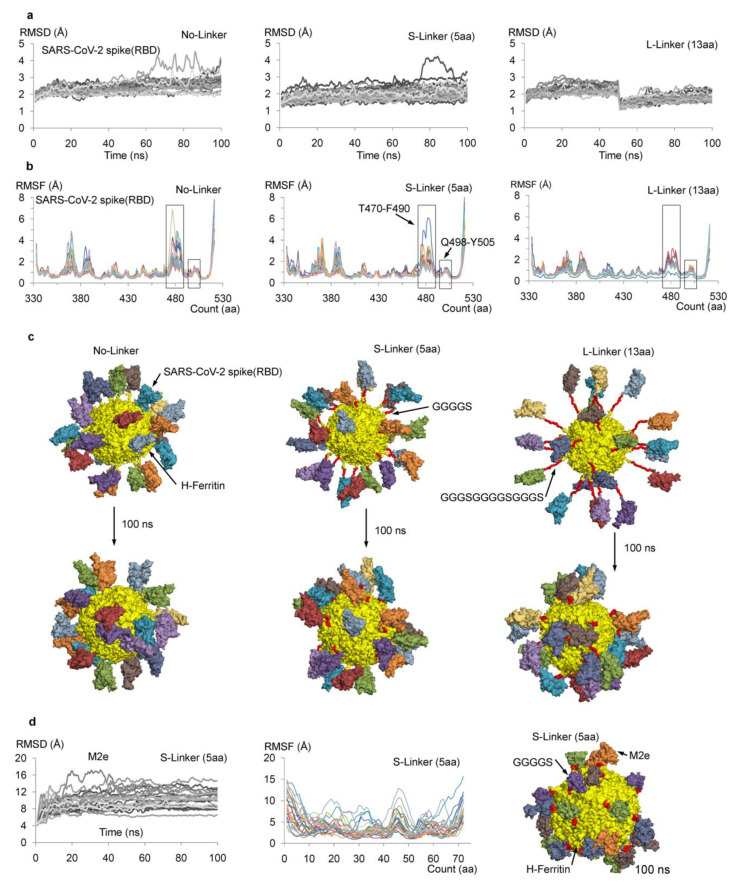
Dynamics of the SARS-CoV-2 spike RBDs when presented over the ferritin nanocage, with characterization and validation of chimeric constructs by molecular dynamics simulations. (**a**) For the individual (total 24) spike RBD monomers over the H-ferritin cage, the root mean square deviation (RMSD) was computed, showing a time-dependent change in non-hydrogen atoms. (**b**) The root mean square fluctuations (RMSF) for each residue based on the Cα atoms from the spike RBDs. Residues 470TEIYQAGSTPCNGVEGFNCYF490 and 498QPTNGVGY505, suggested to bind with the ACE2 receptor, are highlighted [34,35,36]. (**c**)The conformations detected for the SARS-CoV-2 spike RBD-H_ferritin complexes, with different chimeric constructs varying in the length of the linker (No-Linker, 5 aa S-Linker, and 13 aa L-Linker). The protein coordinates were retrieved from the beginning and end of the molecular dynamics simulation. (**d**) The M2e-H_ferritin system (5 aa S-Linker), the conformation dynamics, and the structural properties (RMSD and RMSF of M2e) were further compared with the modeled spike RBD-H_ferritin constructs. The H-ferritin cage is shown as the yellow surface, the linkers are in red, and each monomer of the SARS-CoV-2 spike RBD is represented as the surface in different color.

The secondary structures (α-helices, β-sheets/strands, and loops) of the SARS-CoV-2 spike RBD and the H-/L-ferritin cages can acquire a high degree of freedom in a solvent environment (water and ions) during MDS, and changes in their secondary structures can illustrate the stability of the designed chimeric construct. Hence, an effective means to measure flexibility/stability was applied on the simulated systems using root mean square deviation (RMSD) and root mean square fluctuations (RMSF). Measuring the time-dependent change in non-hydrogen atoms, i.e., the RMSD of 24 individual monomers of SARS-CoV-2 spike RBD and ferritin nanocage proteins, suggested that the ferritin showed a conserved α-helical structure throughout the MD simulations (Figure 2a). By contrast, the spike RBD monomers over the nanocage showed comparatively higher fluctuations (Figure 2a). Retrieving the RMSDs for each monomer of spike RBD over the ferritin cage from the MD time course, a majority (out of 24 monomers) of the spike RBD domains with the No-Linker and 13aa L-Linker were less flexible compared to those with the 5aa S-Linker (Figure 2a). For most of the SARS-CoV-2 spike RBD monomers, the difference between the minimum and the maximum RMSD values was ~1 Å in the No-Linker and the 13 aa L-Linker systems, whereas this RMSD difference was slightly higher at about ~2 Å in the system with the 5 aa S-Linker (Figure 2a).

A standard approach for computing the root mean square fluctuations based on the Cα (c-alpha) atoms for each amino acid from the SARS-CoV-2 spike RBD protein was applied, and the atoms were computed (Figure 2b). The overall comparison of the RMSF from individual spike RBD monomers for all three linker systems (No-Linker, 5 aa S-Linker, and 13 aa L-Linker) illustrated a similar pattern of fluctuation in the residues (Figure 2b). Particularly, the 13 aa L-Linker system had a highly stable spike RBD compared to the other two simulated spike RBD systems. The No-Linker and 5 aa S-Linker systems had similar high peaks of flexibility in their amino acids (Figure 2b). Residues T470-F490 and Q498-Y505, which are suggested to bind with the ACE2 receptor [34,35,36], demonstrated a higher flexibility. Moreover, after correlating these RMSF findings with the RMSD data, the 13 aa L-Linker system had less flexible spike RBD monomers, which gives a clue that there might have been a higher number of intermolecular interactions between the neighboring spike RBD monomers (Figure 2b).

The effects of a diverse set of linkers for the spike RBD–ferritin chimeric construct were examined, the conformational dynamics of each simulated system were visualized (supplementary material Videos S1–S3), and the extracted protein coordinates from the beginning and end of the molecular dynamics are shown in Figure 2c. Similar to these data derived from the RMSD and the RMSF, the spike RBD monomers from the 13 aa L-Linker system were found to interact more with each other (Figure 2c), suggesting that the 13 amino acid-based linker is too long (supplementary material Video S3) for the spike RBD protein to be presented over the surface of a ferritin nanocage. Furthermore, in the No-Linker (supplementary material Video S1) and 5 aa S-Linker (supplementary material Video S2) systems, a higher number of the spike RBD monomers maintained a safe distance on the nanocage; however, the 5 aa S-Linker system had more free spike RBD domains (Figure 2c) compared to the other system (No-linker). These data suggest that the 5 aa (GGGGS) linker was producing an optimum conformation for the spike RBD binding on the ferritin nanocage. Additionally, the 5 aa S-Linker system produced an “up” active state conformation for the majority of the spike RBD monomers (Figure 2c), which is very important for receptor accessibility. These findings correlated with our previous data [35] and other recent studies [3,34,39,54] that showed the spike RBD domains could have two “up” and “down” conformations, which are ACE2-receptor accessible and ACE2-receptor inaccessible states, respectively.

To confirm the characterization and validation for our synthetic SARS-CoV-2 spike RBD–ferritin chimeric nanocages, we used the M2e-H_ferritin (influenza A virus matrix protein 2 ectodomain; M2e) construct as the template structure that was already experimentally validated [24,44]. In our study, we modeled the M2e-H_ferritin complex, followed by an extensive MD simulation (Figure 2d). Analyzing the conformation dynamics of M2e-H_ferritin suggests that the M2e proteins maintained sufficient/optimal distance between each other over the H-ferritin nanocage (Figure 2d and supplementary material Video S4), which likewise correlated with the experimental data [24,44]. Comparing the conformational dynamics of the RMSD and the RMSF data of the M2e monomers (Figure 2d) with the spike RBD monomers (Figure 2c), the RBD domain from the 5 aa S-Linker and the M2e had similar patterns of RMSD jumps (i.e., more fluctuation ranges). Though the spike RBD (333–521 aa) was bigger in size compared to the M2e (1–72 aa) protein, the secondary structure of the spike RBD protein was found to be better characterized relative to the M2e (Figure 2c,d).

**Figure 3:**
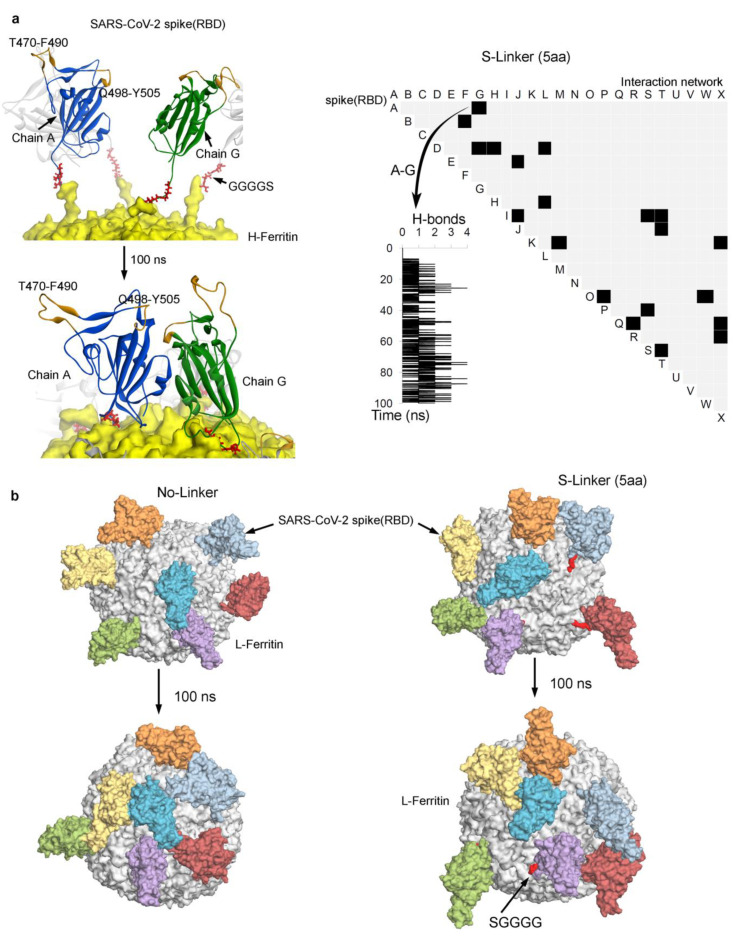
Interaction network between the SARS-CoV-2 spike RBD monomers and the change in their secondary structures over the ferritin nanocage. (**a**) The right panel represents the S-Linker systems and the hydrogen bond (H-bond) intermolecular interactions between the spike RBD monomers observed during the molecular dynamics (MD) time course; each black box describes the binding between two specific spike RBD monomers. As an example, the interaction between the monomers chain A and chain G is presented as a graph plot. Investigating the secondary structures of chain A and chain G (left panel) in the spike RBD monomers suggests that both monomers interact with each other by the end of the MD simulation. Additionally, the spike RBD regions (T470-F490 and Q498-Y505; highlighted in orange) that were proposed to interact with the host ACE2 receptor lacked intermolecular interactions over the ferritin nanocage. The H-bond parameters were 3.5 Å for the donor–acceptor distance and 160°–180° for the intermolecular angle. (**b**) The dynamics of the SARS-CoV-2 spike RBD-L_ferritin systems with two different chimeric constructs with varying lengths of linkers (No-Linker and 5 aa S-Linker) are shown. The protein coordinates were retrieved from the beginning and end of the MD simulations. The H-ferritin cage is shown as the yellow surface, the L-ferritin is grey, the linkers are red, and each monomer of the SARS-CoV-2 spike RBD is represented as a surface/ribbon in different color.

The conformation dynamics of the SARS-CoV-2 spike RBD protein with different chimeric constructs, varying in the length of the linker (No-Linker, 5 aa S-Linker, and 13 aa L-Linker), suggest that the spike RBD monomers in the 5 aa S-Linker system maintained sufficient distance between each other, while maintaining flexibility (that could help to induce ACE2 binding) at the same time. Furthermore, we retrieved the intermolecular hydrogen bond interactions (H-bond; 3.5 Å for the donor–acceptor distance and 160°–180° for the intermolecular angle) between 24 spike RBD monomers over the H-ferritin nanocage for the S-Linker system (Figure 3a). The interaction network presented in Figure 3a (right panel) indicates that the majority of the spike RBD had negligible interactions with other partners over H-ferritin, and that these free spike RBD monomers exhibited a higher probability to interact with the host ACE2 receptor. As an example, the interaction between two spike RBD monomers (chain A and chain G) over the time course of the MD simulation, as well as the secondary structures of proteins, are demonstrated in Figure 3a (left panel). Despite interacting with each other, both monomers (chain A and chain G) maintained “up” receptor accessible conformation (Figure 3a). In addition, regions T470-F490 and Q498-Y505, from the spike RBD protein that was proposed to make interaction with the host ACE2 receptor, were often found free from any intermolecular H-bond interactions over the ferritin nanocage (Figure 3a). Considering these observations, we further monitored the intermolecular H-bond interactions for chain A (spike RBD monomer) with five other surrounded monomers (chains F, G, I, S, and B) for all simulated systems. In the No-Linker system, chain A formed strong interactions with two other monomers (chains F and I), whereas in the L-Linker system, chain A formed interactions with three other monomers (chains B, G, and I).

The MD simulation findings suggest that the five amino acid-based S-Linker is the optimal intermediate length of a linker for the presentation of the spike RBD over H-ferritin. To further check the effectiveness of this S-Linker over other ferritin systems, the L-ferritin was simulated with the S-linker and spike RBD for 100 ns (Figure 3b). Similar to H-ferritin, the L-ferritin nanocage system also demonstrated optimal presentation of the spike RBD with the 5 aa S-Linker system, compared to that of the No-Linker complex. Additionally, a greater number of the spike RBD monomers from the 5 aa S-Linker system were found in the “up” active conformation state, an essential criterion for receptor binding (Figure 3b).

## 4. Conclusions

Nanomaterials, especially protein nanocages (e.g., ferritin), can be extremely useful for vaccine development against antigens such as the spike protein target derived from the SARS-CoV-2 coronavirus. Though smaller in size (H-ferritin cage size has a 12 nm outer diameter) when compared to the COVID-19 viron (a single viron size is ~60–140 nm), these ferritin nanocages can mimic the SARS-CoV-2 coronavirus very effectively if a proper surface protein is incorporated in their system. In this work, we built replicas of several spike RBD-H/L_ferritin constructs with variable linkers (No-Linker, 5 aa S-Linker, and 13 aa L-Linker) and performed extensive molecular dynamics simulations. The secondary structure stability for each monomer of spike RBD protein over the H-ferritin nanocage suggests that the No-Linker and 13aa L-Linker systems were more stable (with the minimum and maximum RMSD differenceat ~1 Å) and had less flexibility compared to the 5 aa S-Linker system (~2 Å RMSD difference). In addition, the conformational dynamics of each simulated system suggest that in the 13 aa L-Linker system, the spike RBD monomers were interacting specifically with each other, and a majority of them lacked the “up” active conformations. In contrast to 13 aa L-Linker, in the 5 aa S-Linker system, a majority of the spike RBDs maintained an optimal distance to each other, resulting in more available free spike RBD for the ACE2 receptor interactions. Additionally, in the case of the 5 aa S-Linker system, a greater number of spike RBD domains maintained “up” active conformational state (receptor accessible), suggesting an optimal intermediate length of the linker. Similar to the H-ferritin, the L-ferritin nanocage showed a well-defined presentation of the spike RBD with only a 5 aa S-Linker. Overall, our findings suggest that a maximal level of “up” active conformations can be obtained from a proper combination of linker rigidity (flexibility) and steric support, due to the inter-spike RBD interactions. Our findings also suggest that the proper linker length for the chimeric constructs can depend on the size of the spike RBD and the inter-spike RBD distance. Based on our comprehensive computational study showing the dynamics of a spike RBD–ferritin nanocage, further experimental validation would determine whether such a chimera could impact future vaccine development against SARS-CoV-2 and related viruses.

## Data Availability

Data is contained within the article or supplementary material.

## Supplementary Materials

The following are available online at https://www.mdpi.com/2218-273X/11/2/297/s1. Video S1: The conformational dynamics of the SARS-CoV-2 spike RBD protein and H-ferritin observed during the MD simulation for the No-Linker system. The H-ferritin nanocage is shown as a silver tube representation, and each monomer of the SARS-CoV-2 spike RBD is in different color. Video S2: The conformational dynamics for the SARS-CoV-2 spike RBD protein and H-ferritin from the 5 aa S-Linker system observed during the MD simulation. The H-ferritin cage is shown in a silver ribbon representation, the linker is in red, and each monomer of the SARS-CoV-2 spike RBD is in different color. Video S3: The conformational dynamics for the SARS-CoV-2 spike RBD protein and H-ferritin from the 13 aa L-Linker system observed during MD simulation. The H-ferritin cage is shown in a silver tube representation, the linker is in red, and each monomer of the SARS-CoV-2 spike RBD is in a different color. Video S4: The conformational dynamics for the M2e protein and H-ferritin from the 5 aa S-Linker system observed during the MD simulation. The H-ferritin cage is shown in a silver representation, the linker is in red, and each monomer of the M2e protein is represented as a tube in a different color.
